# A Holistic Approach to Unravel Keloid Pathogenesis and Optimize Therapeutic Outcomes

**DOI:** 10.3390/biomedicines14040809

**Published:** 2026-04-02

**Authors:** Zhendong Cao, Wei Liu

**Affiliations:** 1 Department of Traditional Chinese Medicine, Shanghai Ninth People’s Hospital, Shanghai Jiao Tong University School of Medicine, Shanghai 200011, China; 18901708559@189.cn; 2 Department of Plastic and Reconstructive Surgery, Shanghai Tissue Engineering Key Laboratory, Shanghai Ninth People’s Hospital, Shanghai Jiao Tong University School of Medicine, Shanghai 200011, China

**Keywords:** keloid, Traditional Chinese Medicine (TCM), holistic approach, pro-inflammatory/fibrotic constitution, combined systemic and local therapies

## Abstract

Although tremendous progress has been made over the past decades in elucidating the mechanisms of keloid formation and developing therapeutic interventions, keloid remains challenging to cure and prone to recurrence. Historically, keloid has been regarded as a benign skin tumor, with mechanistic investigations and therapeutic efforts focused primarily on keloid tissue and cells themselves. In contrast, Traditional Chinese Medicine (TCM) posits that skin diseases manifest from internal bodily dysfunction or dysregulation. Inspired by the holistic principles of TCM, together with the literature reports and clinical evidence of keloid constitution (a systemic condition characterized by an individual’s predisposition to keloid formation), we propose that keloid is likely a pseudo-skin tumor profoundly influenced by its pathological microenvironment. Accordingly, we propose keloid as a persistent inflammation-driven proliferative skin disorder, and elimination of the inflammatory microenvironment may be essential for curing keloid and preventing relapse. Based on this concept, we developed a holistic therapeutic approach that combines systemic treatment targeting keloid constitution with local therapies, including surgery, chemo/radiotherapy, and compression therapy, for the keloid lesion itself. The systemic component encompasses lifestyle and dietary modifications, stress management, physical exercise, as well as the oral administration of TCM herbal medicines and small chemical compounds to suppress systemic inflammatory and fibrotic status, thereby improving keloid constitution. This article introduces this novel holistic approach along with supportive case studies.

## 1. Introduction

Keloid is a fibrotic skin disorder and is also classified as a benign skin tumor. It remains difficult to cure, with recurrence commonly observed following various therapeutic interventions, thus posing a significant challenge in clinical practice. According to currently available literature, conventional strategies for investigating keloid mechanisms and developing therapies have remained largely unchanged: keloid is still regarded as a purely cutaneous disease, with pathogenesis thought to be confined to the skin lesion itself, and all therapeutic approaches target only the local lesion. In contrast, Traditional Chinese Medicine (TCM) views skin diseases (including keloid) as manifestations of internal organ dysfunction or systemic imbalance. Accordingly, ideal treatment should focus on restoring systemic equilibrium (such as balancing *Yin* and *Yang*) and regulating organ functions. Notably, modern medical research has increasingly recognized the critical role of systemic inflammation in keloid pathogenesis [1]. Drawing upon our clinical experience, the existing literature, and TCM theory, we propose a holistic approach to elucidate keloid pathogenesis and optimize therapeutic outcomes.

## 2. Conventional Strategy for Keloid Pathogenesis and Therapy

According to the existing literature, keloids have long been characterized as a form of cutaneous fibrotic disease or a “benign skin tumor”. Although genetic predisposition is recognized as a key systemic pathogenic factor, classic keloid research has traditionally described the pathological changes and underlying mechanisms as confined to the affected skin tissue. These localized factors include alterations in the growth factor milieu, enhanced cell proliferation, genetic mutations, abnormal metabolism of the extracellular matrix (ECM) and collagen, dysregulated local immune responses at lesional sites, and increased wound tension [2,3].

In terms of treatment, both the first [4] and second editions [5,6] of the international scar management guidelines recommend therapeutic strategies that exclusively target the affected keloid area. These include intralesional drug injection, cryotherapy, surgical excision, radiotherapy, laser therapy, and compression therapy. Such longstanding recommendations indicate that the academic community continues to classify keloids as localized skin lesions, with research and therapeutic efforts remaining predominantly focused on the local pathology alone.

## 3. Limitations in Current Strategies of Keloid Mechanism Investigation and Therapy

The term “keloid” (historically spelled “cheloid,” derived from the Greek word *chele*, meaning “crab-like”, due to the lesion’s invasive, claw-like growth pattern) has been part of Western medical literature since the 18th century [7]. Over recent decades, significant advances have been made in understanding keloid pathogenesis and improving therapeutic approaches. These include insights into cellular and molecular pathology, transcriptomic profiling, genetic analyses, and single-cell sequencing [1], as well as clinical interventions such as intralesional injections, cryotherapy, surgical excision, and chemotherapy or radiotherapy [1,2,3,4,5,6]. Collectively, these developments have deepened our understanding of the biological mechanisms underlying keloid formation and have enhanced treatment efficacy and strategies for preventing recurrence.

However, from a clinical standpoint, keloids remain notoriously difficult to cure, and complete prevention of recurrence is still unattainable. This therapeutic challenge arises from two major limitations. First, even in patients with solitary, localized lesions, current treatments sometimes fail to fully suppress lesion growth or prevent relapse after intervention. Second, in individuals with multiple or extensively disseminated keloids, conventional localized therapies are frequently impractical due to the widespread distribution of lesions.

In parallel, research into keloid pathogenesis has largely focused on intrinsic cellular abnormalities, such as hyperproliferation, excessive ECM deposition, aberrant expression of growth factors, dysregulated intracellular signaling pathways, and epithelial–mesenchymal transition (EMT) [1,2,3,4,5,6]. These pathological features, combined with the lesion’s capacity to invade surrounding healthy tissue, have led to the characterization of keloids as “benign skin tumors”. Consequently, anti-tumor strategies have been increasingly adopted in keloid management over the past decades.

Nevertheless, unlike typical skin tumors, no viable animal model of keloids has been successfully established to date. Moreover, in vitro, keloid cells do not behave like tumor cells as they rapidly lose their pathological phenotypes, such as potent proliferative capacity and robust ECM production, after more than three passages [8]. These observations suggest that the pathogenesis of keloids may not depend solely on intrinsic abnormalities of keloid cells themselves; other critical factors are likely involved in disease initiation and progression. Accordingly, the limitations of the current lesion-focused strategy are evident, and a deeper understanding of the true pathogenic mechanisms, as well as efforts to optimize therapeutic outcomes, warrant further investigation.

## 4. TCM Perspective of Skin Disease and Keloid

Driven by rapid advances in science and technology, modern medicine is increasingly investigating disease mechanisms through a reductionist lens, i.e., progressing from general pathology to cellular pathology, molecular pathways, and even single gene-focused mechanisms. Over the past decades, keloid research has followed this trajectory, yielding valuable insights into its pathogenesis. However, this approach may not be sufficient to fully capture the complexity of the disease. For instance, large-scale genome-wide association studies (GWAS) aiming to identify exonic mutations in keloid patients have thus far failed to yield consistent findings [9,10].

Interestingly, ancient Greek medicine posited that health depends on the equilibrium of fundamental bodily constituents (such as the four humors), which are derived from and continuously influenced by the environment [11]. Similarly, Traditional Chinese Medicine (TCM) is characterized by its holistic framework, which seeks to understand disease etiology and formulate treatment strategies by considering the body as an integrated whole, which dynamically interacts with its internal and external environments [12].

According to the TCM theory of visceral manifestation, the health of various tissues throughout the body is closely tied to the functional status of the internal organs. From this perspective, skin disorders are understood not as isolated surface conditions, but as outward manifestations of internal organ dysfunction or systemic imbalance. This holistic view is vividly articulated in the *Huangdi Neijing-Suwen* (Inner Canon of the Yellow Emperor-Plain Questions), which states: “All painful, itchy, and ulcerative skin conditions are associated with the heart (*fire*)”. The rationale is that the heart governs blood and vessels; when pathological “*heart fire*” interacts with blood, it can give rise to ulcerative lesions on the skin [13].

Another illustrative example is the TCM principle that “the lung governs the skin and body hair”. When the lung’s dispersing function (responsible for distributing *Qi*, body fluids, and nutrients) is compromised, it can lead to disruptions in the skin and body hair’s roles in *Qi* diffusion and fluid excretion. Drawing on the classic theory that “the lung and large intestine are internally–externally related”, dysfunction of the large intestine may in turn restrict the physiological activities of the lung, thereby indirectly impairing normal skin function. Furthermore, the *Zhubing Yuanhou Lun* (Treatise on the Causes and Manifestations of Various Diseases) describes: “The spleen governs the muscles; internal heat warms the spleen *Qi* and warmed spleen *Qi* generates *heat* in the muscles. When *dampness* and *heat* intermingle, widespread skin ulcers ensue”. These perspectives converge on the axiom articulated in the *Huangdi Neijing-Lingshu* (Inner Canon of the Yellow Emperor-Miraculous Pivot): “Changes in the interior must manifest externally”, underscoring that skin diseases are, fundamentally, outward expressions of internal bodily imbalances [13].

TCM also associates specific emotional activities with corresponding organ functions, and emotional disturbances can influence the progression of skin diseases by impairing these organs. Well-established concepts such as “grief impairs the spleen” and “sorrow impairs the lung” exemplify this relationship. Additionally, dietary indiscretion constitutes another significant predisposing factor for skin disorders. The *Huangdi Neijing-Lingshu* states that “excessive consumption of rich, greasy, and indulgent foods gives rise to large boils”, highlighting the role of a high-fat, heavy diet in the development of cutaneous boils and furuncles [13].

Actually, keloid was documented in ancient TCM literature under the term “Crab Foot-Like Swelling”. Its pathogenesis is attributed not only to localized skin injury but also to systemic dysfunctions, including *qì xuè yū zhì* (stagnation of *Qi* and *Blood*), *tán shī níng jù* (accumulation and coagulation of *phlegm-dampness*), *dú rè wèi jìn* (incomplete elimination of toxic *heat*), and *bǐng fù bù nài* (constitutional hypersensitivity). This indicates that systemic abnormalities are integral to keloid development, and these identified constitutional imbalances provide a theoretical foundation for TCM interventions targeting the keloid diathesis [14].

## 5. Emerging Evidence of Keloids as a Manifestation of a Systemic Disease

Clinically, severe keloids are frequently observed in patients with a predisposition to keloid formation, commonly referred to as keloid constitution. In a recent TCM investigation conducted by the authors, *Phlegm-Dampness (PD)* or *Dampness-Heat (DH)* constitution, two of the nine recognized TCM constitution types, emerged as predominant phenotypes associated with keloid diathesis. These constitutional profiles are considered analogous to a systemic inflammatory predisposition [15,16].

**Figure 1:**
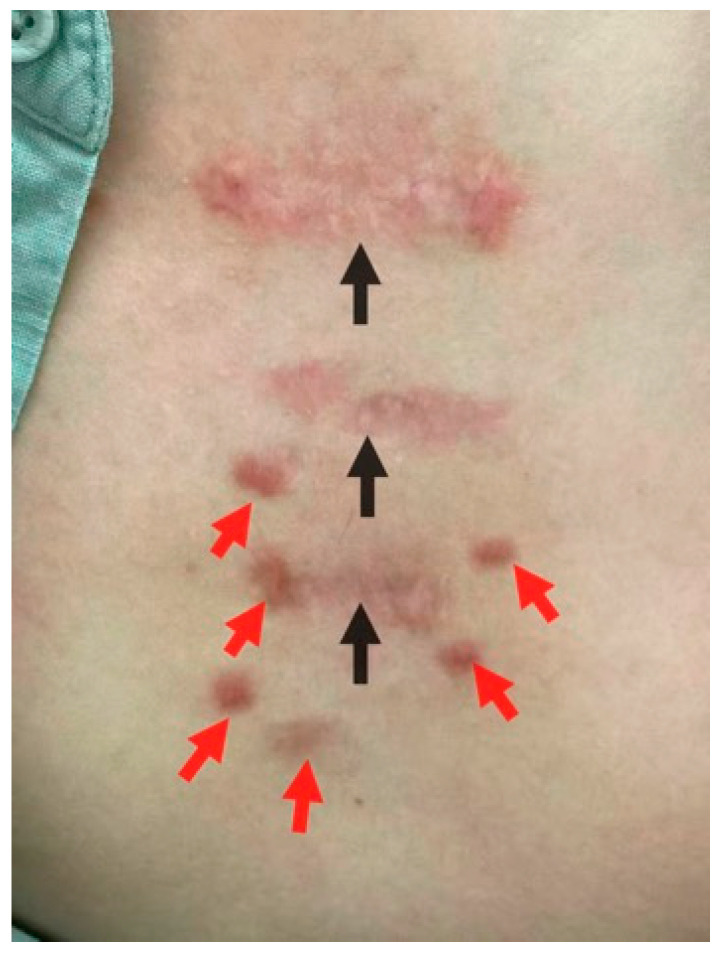
Case 1: A patient with three chest keloids was treated with surgical excision followed by radiotherapy. One year after completion of treatment, the original lesions remained silent with no evidence of recurrence (black arrowed). However, new, scattered small keloid nodules spontaneously developed in the surrounding skin (red arrowed). The picture comes from the physician’s clinical database.

**Figure 2:**
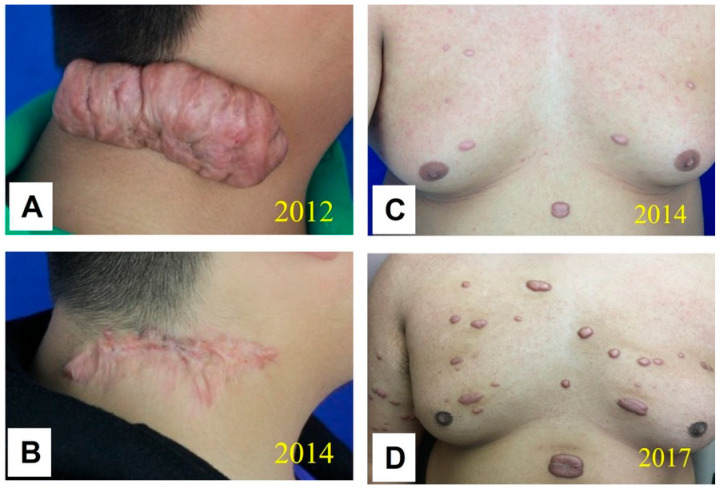
Case 2: A patient presented with a large keloid on the right side of the neck that had recurred after three prior surgical excisions (**A**). The patient subsequently underwent a fourth surgical excision by the author followed by radiotherapy and 12 injections of 5-fluorouracil (5-FU)/corticosteroids (once a month) as well as steroid tape for 20 months post-surgery, which ultimately suppressed recurrence at the original site (**B**). However, concurrent with the control of regrowth at the primary location, new keloid nodules spontaneously developed and spread across the chest (**C**) and other areas. At five years post-surgery, while the original site remained free of recurrence, the disseminated keloid nodules had progressively enlarged (**D**). The picture comes from the physician’s clinical database.

In addition, the authors have observed several clinically intriguing phenomena that suggest systemic involvement in keloid pathogenesis. For instance, in some patients, following complete remission of a large solitary keloid achieved through surgical excision and adjuvant radiotherapy, new scattered small keloid nodules spontaneously emerged around the treated area and progressively increased in size (Case 1, Figure 1). In other cases, after localized keloids were “harshly treated” with surgery, radiotherapy, and sustained intralesional injections, isolated small keloid nodules unexpectedly developed at sites distant from the original lesion (Case 2, Figure 2). These observations imply that certain systemic pathogenic factors may “redistribute” to alternative cutaneous sites when the original lesion was rendered unresponsive to their pathological influence by localized radiotherapy. Given that keloid-derived fibroblasts, unlike malignant tumor cells, lack metastatic capacity, these clinical findings strongly suggest that systemic pathogenic factors constitute an indispensable component of keloid etiology.

Indeed, a growing body of well-established and emerging evidence supports the involvement of systemic factors in keloid development. First, familial inheritance is a well-characterized systemic determinant, typically following an autosomal dominant pattern with incomplete penetrance, variable expressivity, and delayed dominance [17]. Endocrine hormones constitute a second confirmed systemic factor, as keloids most frequently arise during adolescence and tend to regress with advancing age. Their size often increases markedly during pregnancy and growth slows following menopause [18]. In adolescent patients, those with acne-induced keloids exhibit elevated systemic levels of androgens and androgen receptor expression, further corroborating the role of hormonal influence [19].

Dietary influences represent another clinically observable systemic factor. Alcohol consumption, particularly of strong liquor, is frequently observed to exacerbate pruritus and stinging sensations in keloids and may accelerate disease progression. Some patients experience symptom exacerbation after ingesting spicy foods such as chili peppers or “heat-inducing” foods (e.g., mutton). In a subset of patients, seafood intake also aggravates symptoms, potentially in association with food allergies. Furthermore, studies have reported that African keloid patients tend to have a higher dietary intake of omega-6-enriched fatty foods [20,21]. Indeed, unhealthy lifestyle factors [22], including poor dietary patterns [23], sleep disturbances [24], and psychological stress [25], have been implicated in keloid progression [26], providing further evidence for the contribution of systemic factors to keloid development. In addition, hypertension [27] and dysregulation of the renin–angiotensin system (RAS) [28] may represent additional systemic determinants.

## 6. What Is Likely the True Nature of Keloid?

For a long time, keloids have been characterized as “benign tumor”-like skin lesions. However, it is clinically unusual for benign skin tumors of the same etiology to exhibit widespread distribution across the body or to recur following complete surgical excision, suggesting that keloids are unlikely to be true skin tumors arising from clonal expansion of genetically mutated cells [8]. Several lines of evidence further underscore the fundamental distinction between keloids and conventional benign skin tumors:

(1) Keloids can recur indefinitely after surgical excision, regardless of the completeness of resection, in the absence of adjunctive interventions such as radiotherapy or steroid injection.
(2) No validated animal model of keloids currently exists, and implantation of keloid-derived cells into immunodeficient animals fails to generate keloid-like lesions.
(3) Isolated keloid fibroblasts lose their pathological phenotype, including elevated proliferative capacity and robust extracellular matrix production, after more than three passages in vitro.
(4) Keloid cells require a specific pathological microenvironment to survive and to exert their pathogenic effects, a condition often referred to as keloid constitution that is significantly modulated by lifestyle factors such as dietary patterns and daily habits.

Collectively, these observations suggest that keloid may represent an induced, pseudo-tumor-like lesion whose development and persistence are critically dependent on a permissive pathological microenvironment, specifically, the keloid constitution. Composed of highly proliferative keloid fibroblasts and an abundant extracellular matrix, this pseudo-tumor is, in fact, driven by an inflammatory milieu. It therefore aligns with the definition of a fibro-proliferative disorder or reactive hyperplasia. In light of accumulating evidence pointing to an inflammatory microenvironment within keloid tissue [29], we propose redefining keloids as a persistent inflammation-driven proliferative skin disorder, and eliminating the inflammatory milieu is essential not only for effective treatment but also for preventing post-therapeutic recurrence.

When such an inflammatory microenvironment persists, it may progressively reprogram normal skin cells into dysfunctional keloid phenotypes through epigenetic dysregulation [30,31] or somatic mutations (e.g., in p53 or Fas ligand) [32,33]. Thus, the pathogenic microenvironment may serve as a primary driver for keloid initiation and progression.

## 7. Systemic Pro-Inflammatory State May Be a Key Component of Keloid Constitution

Although keloids exhibit a degree of genetic predisposition, the majority of patients seeking medical care report no clear family history of the condition. Moreover, two GWAS conducted on blood cells of keloid patients failed to identify definitive pathogenic mutations within exon regions [9,10]. In contrast, epigenetic dysregulation has emerged as a likely central mechanism in keloid pathogenesis [34,35]. Previously, our group investigated epigenetic differences between keloid tissues and their surrounding normal skin using RNA-Seq analyses of mRNA, miRNA, and lncRNA. We identified over 3700 differentially expressed mRNAs, more than 3000 differentially expressed long non-coding RNAs, and over 270 differentially expressed microRNAs between keloid and adjacent normal skin, highlighting a substantial epigenetic contribution to keloid pathogenesis. Furthermore, KEGG analysis revealed significantly altered signaling pathways between the two tissue types, including Wnt signaling, regulation of stem cell pluripotency, PPAR signaling, and inflammatory mediator pathways. Elucidating the specific mechanisms linking environmental factors to epigenetic dysregulation represents a compelling direction for future research into keloid pathogenesis [36].

Epigenetic alterations are increasingly recognized as the primary molecular interface through which environmental factors, such as dietary habits [37], psychological stress [38], and biorhythm disruption [39], exert their pathological effects. Notably, each of these factors is also implicated in the development of chronic systemic inflammation [40].

Indeed, a growing body of evidence implicates persistent systemic inflammation as a key component of keloid pathogenesis [1]. In a comparative study involving 84 keloid patients and healthy controls, circulating levels of granulocyte colony-stimulating factor (G-CSF), granulocyte-macrophage colony-stimulating factor (GM-CSF), and interleukins 4, 6, and 13 were found to be significantly elevated in the keloid group [41]. Elevated serum IL-6 levels in keloid patients have been corroborated by multiple independent studies, while an inverse association between plasma IL-37 levels and keloid severity has also been reported [1]. IL-17 may further contribute to the keloid inflammatory microenvironment, potentially via the IL-17/IL-6 axis [42,43].

Further evidence supports the association between immunoglobulin profiles, atopic predisposition, and keloid pathogenesis. Across diverse ethnic and age groups, keloid severity has been positively correlated with serum immunoglobulin E (IgE) levels [44]. Moreover, keloid patients exhibit a higher prevalence of allergic reactions compared to those with hypertrophic scars [45]. Relative to healthy controls, individuals with keloids demonstrate significantly elevated serum levels of immunoglobulin M (IgM) and immunoglobulin G (IgG), alongside markedly reduced immunoglobulin A (IgA) [46].

## 8. Systemic Inflammatory Constitution Increases Keloid Susceptibility and Aggravates Local Inflammatory Responses

Systemic inflammatory constitution, or systemic inflammatory predisposition, represent a persistent systemic inflammatory body condition [15], which have become the comorbidity for various diseases. Keloid constitution could be considered as analogous to it. Under normal conditions, minor skin injury triggers a transient inflammatory response that recruits immune cells for antimicrobial defense and wound debridement. This response is usually self-limiting and typically subsides without sequelae. However, in individuals with a keloid constitution, likely a state of low-grade persistent systemic inflammation driven by factors, such as metabolic syndrome, poor diet, chronic stress, biorhythm disruption, and autoimmune conditions, even minor skin injury or folliculitis can prime dermal fibroblasts. This occurs through the recruitment of circulating inflammatory cells into the lesional site. Once primed, these fibroblasts become hyperactivated within the inflammatory microenvironment, proliferating rapidly and depositing excessive ECM. This process leads to the formation of keloid tissue, which autonomously produces cytokines and growth factors that perpetuate local pathology.

Consequently, the active keloid contributes additional inflammatory mediators into the systemic circulation, reinforcing or exacerbating the preexisting systemic inflammatory state. This amplified systemic inflammation, in turn, primes a broader population of fibroblasts, both locally and distally. Such a self-perpetuating cycle drives the progressive enlargement of existing keloids and facilitates the formation of new keloids at sites of even trivial trauma. It also underpins the clinical challenges in achieving durable treatment responses and preventing recurrence, as the underlying systemic inflammatory diathesis remains active.

Therefore, we propose that the investigation of keloid pathogenesis requires a fundamental paradigm shift, from viewing keloids as localized scars to understanding them as the product of the dynamic interplay between systemic inflammation and susceptible skin cells. Critically, this reconceptualization calls for a holistic therapeutic strategy that concurrently addresses both the systemic inflammatory milieu and the localized keloid lesion.

## 9. A Holistic Therapeutic Approach to Enhance Keloid Curing and Prevent Its Relapse via Combined Systemic and Local Treatments

Building on the understanding that systemic inflammation may contribute significantly to keloid pathogenesis, the authors adopted a holistic therapeutic approach aimed at both treatment and relapse prevention. We began by identifying potential risk factors that may promote or sustain systemic inflammation. Among patients with severe keloids, particularly those presenting with widely disseminated nodules, several common clinical features emerged, including:

(1) Biorhythm disruption due to habitual late sleep, often past midnight or until 1–3 AM accompanied by persistent fatigue and lack of energy.
(2) Excessive intake of pro-inflammatory foods [47], including high glycemic index carbohydrates such as refined grains, sugary beverages (e.g., soda, sprite, sweetened milk tea), and high-sugar fruits, as well as frequent consumption of fried foods and omega-6-enriched fats.
(3) Constipation, frequently observed in patients with spreading keloids secondary to acne, likely linked to gut dysbiosis and absorption of inflammatory toxins [48].
(4) Psychological stress and physical inactivity, both commonly noted in patients with severe keloids.

Addressing these modifiable risk factors constitutes a core component of the authors’ therapeutic strategy for both keloid treatment and relapse prevention. Prior to and during local intervention, all patients are advised to adopt the following lifestyle modifications:

(1) Maintain a consistent sleep schedule by retiring before 11:00 p.m., and take active measures to ensure adequate sleep quality.
(2) Avoid pro-inflammatory foods and beverages, particularly refined carbohydrates and sugary drinks. Instead, intake of low-glycemic-index carbohydrates and omega-3-rich foods (e.g., deep-sea fish) is encouraged. Spicy foods and alcohol are typically restricted during active disease and the post-operative periods. Anti-inflammatory vegetables, such as spinach, broccoli, and purple cabbage, are recommended. Adequate dietary fiber intake is emphasized to prevent constipation and thereby reduce systemic inflammatory burden.
(3) Incorporate regular physical exercise and stress-relief practices as adjunctive anti-inflammatory measures [49].

In addition to these preventive measures, we employed TCM herbal medicine to treat severe keloid patients presenting with a pronounced *Phlegm-Dampness* or *Dampness-Heat* constitution, using the formula *Jiàn pí qù shī* (Fortify the Spleen and Dispel Dampness). This formular was designed for strengthening the spleen and eliminating dampness, which was modified based on *Zhizhu Pills* (mainly fructus aurantia, rhizoma atractylodis macrocephalae), the *Huanglian Wendan Decoction* (mainly coptis chinensis, bamboo shavings, immature bitter orange, pinellia ternate, dried tangerine, licorice root, fresh ginger, poria cocos), and *Fuzi Lizhong Pills* (mainly prepared aconite root, dried ginger, codonops) [50]. Patients presenting with either severe, large solitary keloids or widespread systemic keloids with confirmed *Phlegm-Dampness* or *Dampness-Heat* constitution were enrolled in the study. Participants received consistent systemic herbal treatment for a period of 1 to 3 months. The treatment protocol followed a sequential approach: patients first underwent constitutional therapy to address the underlying inflammatory/fibrotic tendency. Once systemic improvement was observed, local therapy was subsequently administered. This herbal intervention aims to correct the underlying constitutional imbalance by enhancing visceral function, restoring harmonious inter-organ relationships, and rebalancing fundamental physiological elements such as *Yin* and *Yang* as well as *Qi* and *Blood*. Through this holistic regulatory mechanism, systemic *Dampness* and *Dampness-Heat* are progressively resolved, thereby reducing systemic inflammation. In parallel, systemic anti-inflammatory and anti-fibrotic agents have also been explored as adjunctive strategies for managing keloid constitution.

**Figure 3:**
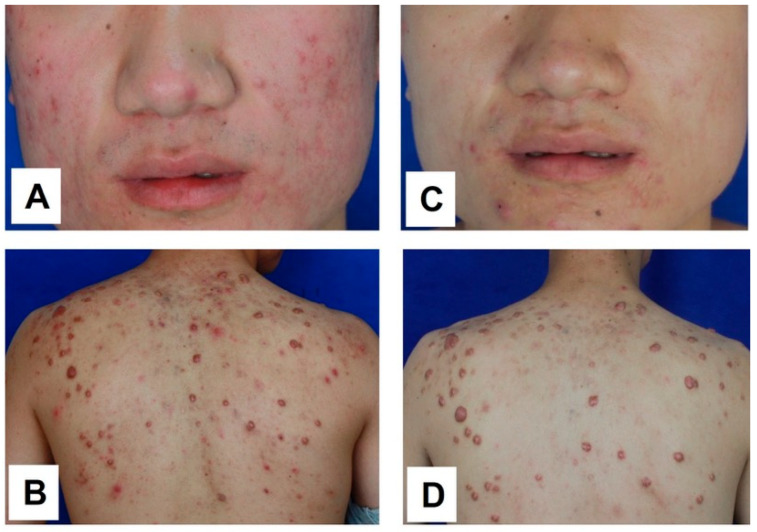
Case 3: A patient presented with severe acne on the face (**A**) and spreading keloid nodules secondary to acne on the trunk (**B**), accompanied by chronic constipation. The skin lesions were characterized by pronounced vascularity and raised fibrotic tissue. Following five months of TCM herbal treatment targeting a dampness-heat constitution, the facial acne resolved and normal skin appearance was restored (**C**), alongside resolution of constipation. After one year of continued herbal therapy, progression of the spreading keloids was effectively halted, and partial or complete regression was observed in some smaller nodules (**D**). The picture comes from the physician’s clinical database.

Case 3 illustrates how systemic constitution may contribute to keloid formation and how constitution-targeted treatment can support keloid resolution and prevent relapse. The patient presented with severe facial acnes (Figure 3A) and spreading keloid nodules secondary to acnes on the trunk (Figure 3B), accompanied by severe constipation (discharge once every three days), a condition likely contributing to systemic inflammation. To specifically address the underlying systemic imbalance, the patient received TCM herbal monotherapy without any concurrent local or procedural interventions.

After five months of herbal treatment, congestive and scattered facial acne lesions had substantially subsided, with most lesions resolving and normal facial appearance restored (Figure 3C). Notably, bowel function normalized completely, with regular discharge twice daily. After one year of continued herbal therapy, progression of the spreading keloid nodules on the back had halted, accompanied by marked reduction in erythema. Interestingly, some smaller nodules had partially or completely regressed, while the remaining keloids remained quiescent, with no evidence of regrowth or recurrence (Figure 3D).

**Figure 4:**
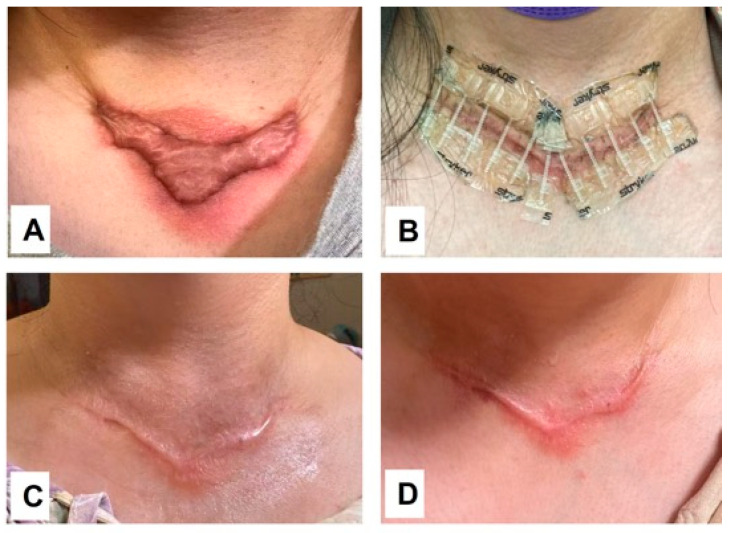
Case 4: A patient presented with a keloid on the lower neck accompanied by severe perilesional erythema and symptoms of itching and stinging (**A**). The patient underwent surgical excision and radiotherapy, combined with oral methotrexate (15 mg once weekly) for three months. At 7 days post-surgery, the erythema and associated symptoms had completely resolved (**B**), and this improvement was largely sustained through the three-month follow-up (**C**). Two months after discontinuation of methotrexate, mild erythema recurred; however, keloid relapse remained well controlled (**D**). The picture comes from the physician’s clinical database.

As proposed, persistent systemic inflammation may play a contributory role in keloid pathogenesis. Direct evidence supporting this concept would be the observed therapeutic efficacy of systemic anti-inflammatory intervention, as demonstrated in Case 4. A female patient presented with a rapidly growing keloid at the lower neck following thyroidectomy. The lesion was characterized by pronounced perilesional erythema, accompanied by significant pruritus and stinging, the clinical features indicative of an active local inflammatory response (Figure 4A). To investigate whether this presentation was driven by an underlying systemic inflammatory constitution, the patient was treated with oral methotrexate (15 mg once weekly) for three months, in conjunction with surgical excision and postoperative electron beam irradiation. Throughout the treatment period, the patient was closely monitored for potential adverse effects via regular complete blood counts and liver and kidney function tests, all of which remained within the normal limits.

Notably, within one week of methotrexate initiation, the perilesional erythema resolved completely (Figure 4B). This therapeutic effect was largely sustained over the first three months, with complete resolution of itching and stinging symptoms (Figure 4C). However, at five months post-surgery, i.e., two months after methotrexate discontinuation, erythema recurred, accompanied by mild symptoms and early hypertrophic changes along the surgical incision line. Importantly, the erythema did not regain its original severity, and the recurrence remained effectively controlled (Figure 4D). This case provides compelling evidence that systemic inflammation, reflective of keloid constitution, is actively involved in keloid initiation, progression, and recurrence. It further underscores the potential value of integrating safe and effective systemic anti-inflammatory and anti-fibrotic therapy into a holistic treatment regimen aimed at achieving sustained keloid resolution and preventing relapse.

**Figure 5:**
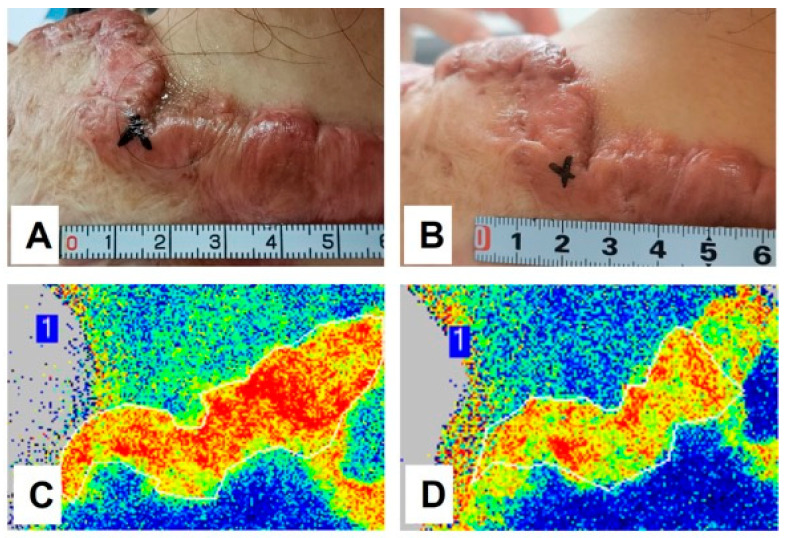
Case 5: A female patient presented with a large, long-standing keloid on her left shoulder, accompanied by marked erythema and symptoms of pain and itching (**A**). She received oral asiaticoside treatment for six months, which resulted in a notable reduction in scar thickness (from 5.68 mm to 4.68 mm measured by ultrasound) and erythema, accompanied by significant symptomatic improvement (**B**). The treatment also led to a substantial decrease in keloid vascularity, as measured by laser speckle contrast imaging (from 180 units, (**C**), to 153 units, (**D**)). Note, laser speckle contrast imaging (LSCI) is a powerful, non-invasive optical imaging technique used primarily to visualize and measure blood flow in tissue. The picture comes from the physician’s clinical database.

According to the literature, currently available systemic agents with anti-fibrotic and anti-inflammatory properties for keloid management include asiaticoside [51] and tranilast [52]. The authors’ group has conducted a clinical trial investigating oral asiaticoside for targeting keloid constitution. The findings confirmed its efficacy in alleviating pain and pruritus, as well as reducing lesional erythema in a subset of patients after six months of treatment (manuscript in preparation), as demonstrated in Case 5. Objective assessments revealed reduced keloid thickness evaluated and measured by ultrasound (Figure 5A,B), and decreased erythema by laser speckle contrast imaging (Figure 5C,D).

Most importantly, the improved keloid constitution paves the way for the effective local treatment of keloid lesions and better control of the relapse. Case 6 illustrates the effectiveness of such an integrative therapeutic approach.

The patient presented with widespread keloid nodules involving the chest, back, bilateral shoulders, and arms. Prior conventional local therapies, including intralesional steroid/5-FU injections, laser therapy, topical steroid tape, surgical excision, and radiotherapy, had all failed with recurrent keloid nodules and continued progression. Subsequently, a holistic treatment strategy was implemented by incorporating lifestyle modifications, oral asiaticoside therapy, and combined local treatments. Specifically, the patient was advised to restrict carbohydrate intake and avoid staying up late. This integrative approach resulted in successful disease control.

**Figure 6:**
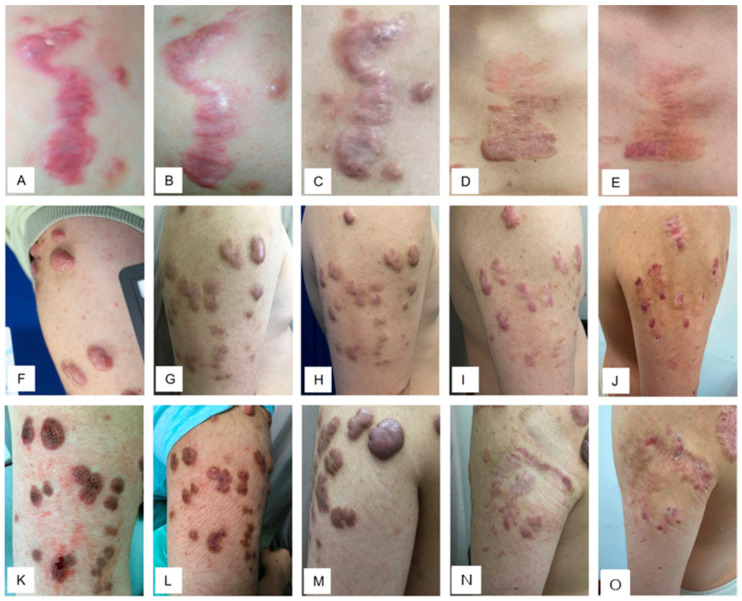
Case 6: A patient presented with widespread keloids involving the chest, bilateral shoulders, arms, and back was successfully treated using combined systemic and local therapies. Briefly, a recurrent chest keloid following surgery and radiotherapy/chemotherapy (**A**) was treated with oral asiaticoside for four weeks, resulting in reduced vascularity (**B**), but the growth persisted even with prolonged oral treatment (**C**). Following two years of combined therapy of oral asiaticoside and topical steroid tape, the lesion became inactive and flattened without recurrence (**D**), and remained stable after an additional six years of steroid tape alone (**E**). Similarly, recurrent keloids on the right arm after surgery and radiotherapy/chemotherapy (**F**) showed reduced erythema following systemic treatment (**G**). Combined therapy with oral asiaticoside and topical steroid tape led to progressive improvement at one year (**H**) and two years (**I**), with complete flattening achieved after an additional six years of tape application and no signs of recurrence (**J**). In contrast, recurrent keloids on the left arm following laser therapy (**K**) and intralesional 5-FU/steroid injections (**L**) continued to enlarge despite reduced vascularity from asiaticoside treatment (**M**). These lesions were surgically excised without adjunctive radiotherapy. Under continued systemic asiaticoside therapy for two years, combined with 12 monthly postoperative intralesional injections of 5-FU/steroid at the surgical site and topical steroid tape application, no recurrence was observed at 20 months postoperatively (**N**). Relapse-free status was maintained throughout an additional six years of follow-up with steroid tape alone (**O**). The picture comes from the physician’s clinical database.

As shown, surgical excision of a chest keloid followed by radiotherapy resulted in recurrence accompanied by severe erythema, itching, and pain (Figure 6A). After four weeks of oral asiaticoside treatment, both the erythema and associated symptoms showed notable improvement (Figure 6B). Continued oral therapy further reduced lesional vascularity, rendering the nodules darker in color; however, it failed to suppress their progressive growth (Figure 6C). Adjunctive topical steroid tape was then applied to the nodules.

Notably, while prior application of steroid tape without systemic treatment had led to site intolerance, manifesting as severe erythema, pain, itching, and epidermal breakdown; no such adverse reactions occurred when the tape was used concomitantly with systemic therapy. Under this combined regimen, the treated nodules gradually flattened and softened over two years (Figure 6D). Steroid tape application was continued for an additional six years alongside maintained lifestyle modifications, without further asiaticoside treatment. The keloids remained flattened with no evidence of recurrence throughout follow-up (Figure 6E).

Similarly, a keloid recurred after surgical excision on the right upper arm, accompanied by increased vascularity (Figure 6F). Following oral asiaticoside treatment, vascularity significantly decreased; however, the nodules continued to enlarge (Figure 6G). Combined therapy with asiaticoside and topical steroid tape led to progressive improvement after one year (Figure 6H) and two years (Figure 6I) of treatment. Steroid tape application was maintained for an additional six years alongside continued lifestyle modifications, with no other interventions. As shown, the previously active nodules became completely softened and flattened, with no evidence of recurrence (Figure 6J).

On the left arm, laser therapy (Figure 6K) and intralesional steroid/5-FU injections (Figure 6L) failed to achieve satisfactory outcomes, with the keloid recurring rapidly following treatment. The nodules enlarged progressively despite a significant reduction in vascularity and a darker clinical appearance (Figure 6M). These lesions were subsequently excised without adjunctive radiotherapy. Notably, under systemic treatment and an improved keloid constitution, regular monthly intralesional injections of 5-FU/steroid for 12 months (once a month) and steroid tape were sufficient to prevent the recurrence through 20 months post-surgery (Figure 6N). The patient was further maintained on topical steroid tape as needed, without systemic therapy or additional interventions, alongside continued lifestyle modifications. As shown, no recurrence has been observed during eight years of follow-up even without radiotherapy (Figure 6O). This case again provides compelling evidence supporting a holistic approach as an ideal strategy for achieving sustained keloid resolution and preventing long-term relapse.

While the approach proposed in this manuscript holds promise, it is not without limitations that require further investigation. First, the hypothesis is currently supported by representative clinical cases; however, more robust evidence from randomized or controlled trials is needed to validate constitution-targeted therapy. Second, the social management component involves public health considerations, where recall bias in lifestyle and dietary histories is likely to exist. Moreover, integrating lifestyle changes, dietary and psychological modifications, and the administration of therapeutic agents into a complex intervention presents a significant challenge in determining the specific role of each factor. A well-designed study incorporating rigorous statistical analysis, biomarker evaluation, and long-term follow-up may offer quantifiable and objective evidence to address this complexity.

Third, a confirmed link between systemic constitutional changes and alterations in keloid characteristics—such as symptoms, vascularity, stiffness, and thickness—as well as their impact on treatment efficacy and relapse prevention, remains to be established. Fourth, understanding the working mechanisms of herb–drug interactions, defining how different herbs in a particular formula work together, and building a possible roadmap for their complex mechanisms within the human body as well as monitoring their distribution and safety in vivo would certainly be beneficial for the future application of TCM in keloid treatment.

## 10. Strategic Innovations in Future Keloid Research

To deepen the understanding of keloid pathogenesis and advance therapeutic development, identifying appropriate research directions is essential.

At the cellular and molecular levels, interactions among different cell types may be the key to understanding how a pathological microenvironment, such as persistent inflammation, can induce fibroblasts and keratinocytes to adopt a keloid phenotype, and sustain or enhance their aberrant behavior. In vitro co-culture systems offer a valuable approach to investigate these dynamics, for example, by co-culturing fibroblasts with macrophages or lymphocytes, or by exploring the roles of inflammatory mediators such as IL-6 and IL-17.

A more refined focus involves elucidating the mechanisms through which microenvironmental factors drive cellular dysfunction, particularly via epigenetic dysregulation or the induction of somatic mutations. A critical goal is to establish a direct link between specific pathological conditions and the epigenetic alterations observed in keloid cells and tissues, such as distinct DNA methylation patterns, histone modifications, and non-coding RNA expression.

Importantly, applying cancer biology strategies to investigate keloid mechanisms would be misleading, as keloids do not arise from intrinsic cellular abnormalities such as the clonal expansion of genetically mutated cells.

Developing a robust in vivo model for keloids is difficult because animals lack the physiological predisposition to form these lesions. Against this background, a promising approach is to engineer a relevant pathological milieu in animal hosts to facilitate the engraftment and growth of implanted human keloid cells or tissue. This could involve inducing a pro-inflammatory systemic environment via drug administration, metabolic or nutritional imbalances, or through the transplantation of keloid patient-derived gut microbiota and cytokine injections.

Clinically, the true nature of keloid constitution and its working mechanism remain to be explored and defined such as evidence of systemic inflammation/systemic fibrosis and their potential sources. Furthermore, how to effectively treat the keloid systemic pathogenic condition to truly implement etiological treatment would be the most important issue. With the removal of etiological risk factors, combined local treatment using current available therapy will most likely cure keloids and resolve the recurrence issue.

In this context, well-designed randomized controlled trials and multicenter studies are imperative to generate robust data and strengthen the conclusions drawn in this article. Furthermore, concrete therapeutic proposals must be developed to translate these insights into feasible and safe systemic treatment strategies.

## 11. Conclusions

Antiproliferative and anticancer approaches have been the primary therapeutic strategies for keloids over the past few decades. However, achieving sustained resolution and the long-term prevention of recurrence remains a significant clinical challenge. In contrast, the holistic perspective of Traditional Chinese Medicine (TCM) offers novel insights into both the underlying mechanisms of keloid formation and the development of more effective treatments. Notably, the concept of “keloid constitution” is a commonly observed clinical phenomenon, analogous to an inflammatory constitution. Drawing upon TCM theory, the existing literature, and the authors’ own clinical experiences, we propose that keloids is likely to be a persistent inflammation-driven proliferative skin disorder, and a holistic approach will be an ideal way to explore the mechanism and develop effective therapy for keloid disorder.

## Data Availability

The original contributions presented in this study are included in the article. Further inquiries can be directed to the corresponding author.

## Abbreviations

The following abbreviations are used in this manuscript:

TCM	Traditional Chinese Medicine
PD	Phlegm-Dampness
DH	Dampness-Heat
IL-	Interleukin
